# mDia1 regulates breast cancer invasion by controlling membrane type 1-matrix metalloproteinase localization

**DOI:** 10.18632/oncotarget.7429

**Published:** 2016-02-17

**Authors:** Daehwan Kim, Jangho Jung, Eunae You, Panseon Ko, Somi Oh, Sangmyung Rhee

**Affiliations:** ^1^ Department of Life Science, Chung-Ang University, Seoul 06974, Korea

**Keywords:** breast cancer cells, invasion, mDia1, membrane type 1-matrix metalloproteinase (MT1-MMP), microtubule dynamics

## Abstract

Mammalian diaphanous-related formin 1 (mDia1) expression has been linked with progression of malignant cancers in various tissues. However, the precise molecular mechanism underlying mDia1-mediated invasion in cancer cells has not been fully elucidated. In this study, we found that mDia1 is upregulated in invasive breast cancer cells. Knockdown of mDia1 in invasive breast cancer profoundly reduced invasive activity by controlling cellular localization of membrane type 1-matrix metalloproteinase (MT1-MMP) through interaction with microtubule tracks. Gene silencing and ectopic expression of the active form of mDia1 showed that mDia1 plays a key role in the intracellular trafficking of MT1-MMP to the plasma membrane through microtubules. We also demonstrated that highly invasive breast cancer cells possessed invasive activity in a 3D culture system, which was significantly reduced upon silencing mDia1 or MT1-MMP. Furthermore, mDia1-deficient cells cultured in 3D matrix showed impaired expression of the cancer stem cell marker genes, CD44 and CD133. Collectively, our findings suggest that regulation of cellular trafficking and microtubule-mediated localization of MT1-MMP by mDia1 is likely important in breast cancer invasion through the expression of cancer stem cell genes.

## INTRODUCTION

Metastasis due to tumor cell migration and invasion is the most common cause of cancer-related deaths. Cytoskeletal proteins and microtubule reorganization are critical for the invasive and migratory activities of metastatic cancer cells [1, 2]. In addition, cancer cell invasion requires proteolytic activity to degrade the extracellular matrix (ECM) components, which could be regulated by cytoskeletal proteins [3, 4]. Thus, disruption of the cytoskeletal network frame may be useful for cancer treatment.

The formin family of proteins is essential for controlling cytoskeletal remodeling [5, 6]. Specifically, mammalian diaphanous-related formin 1 (mDia1) is a potent regulator of actin polymerization and microtubule stabilization [7, 8]. It can act as an upstream regulator of extracellular signal-regulated kinases (ERKs), myosin light chains (MLCs), and p38 mitogen-activated protein kinases (MAPKs) [9]. In addition, mDia1 interacts with cellular Src (c-Src), to guide its localization to the membrane, which subsequently results in Rac activation and membrane ruffle formation through the phosphorylation of breast cancer anti-estrogen resistance protein 1 (also known as p130Cas) [10, 11]. Furthermore, mDia1 can interact with viral Src (v-Src), mediating its movement to the cell periphery and promoting cell invasion and tumorigenesis [12]. This finding suggests that elevated expression of mDia1 in tissues or cells leads to enhanced tumorigenesis and cell invasion.

Recent evidence suggests that cancer cell invasion requires the invadopodia structure, in which diaphanous-related formin (DRF) proteins, including mDia1, are highly enriched along with membrane type 1-matrix metalloproteinase (MT1-MMP). Specially, MT1-MMP confers proteolytic activity to the ECM surrounding the invadopodia structure, resulting in cancer cell invasion [13, 14]. As the individual inhibition of each DRF protein or MT1-MMP decreases invadopodia-mediated ECM degradation and cancer invasion, it seemed that these proteins might be functionally connected for invadopodia-mediated cancer invasion.

Rab8, a small guanosine triphosphatase (GTPase), is implicated in the intracellular trafficking of MT1-MMP and delivering it to the cell surface [15], through a process that depends on the microtubule track [16]. Although the microtubule network and its motor protein, kinesin, are important for the intracellular trafficking of MT1-MMP, the detailed molecular mechanism of how MT1-MMP moves on microtubules has not been fully elucidated. Since DRF proteins are implicated in the regulation of microtubule stabilization and endosomal dynamics [17], mDia1 might possibly contribute to the intracellular transport of MT1-MMP via microtubule track. However, direct evidences for molecular interaction between mDia1 and MT1-MMP on invadopodia function have not yet been reported.

In this study, we found that mDia1 is involved in the intracellular trafficking of MT1-MMP localization to the plasma membrane through the microtubule track. Although overexpression of mDia1 in cancer cells did not change the expression level of MT1-MMP, localization of MT1-MMP to the plasma membrane was significantly impaired by down-regulation of mDia1, possibly due to decreased binding affinity of MT1-MMP with microtubules. Furthermore, we also found that silencing mDia1 in cells impaired their invasive phenotype and expression of cancer stem cell (CSC) markers in a 3-dimensional (3D) collagen matrix system. Our results suggest that overexpression of mDia1 in breast cancer may contribute to increase its invasiveness via MT1-MMP localization on the plasma membrane.

## RESULTS

### mDia1 controls invasion of breast cancer cells

**Figure 1:**
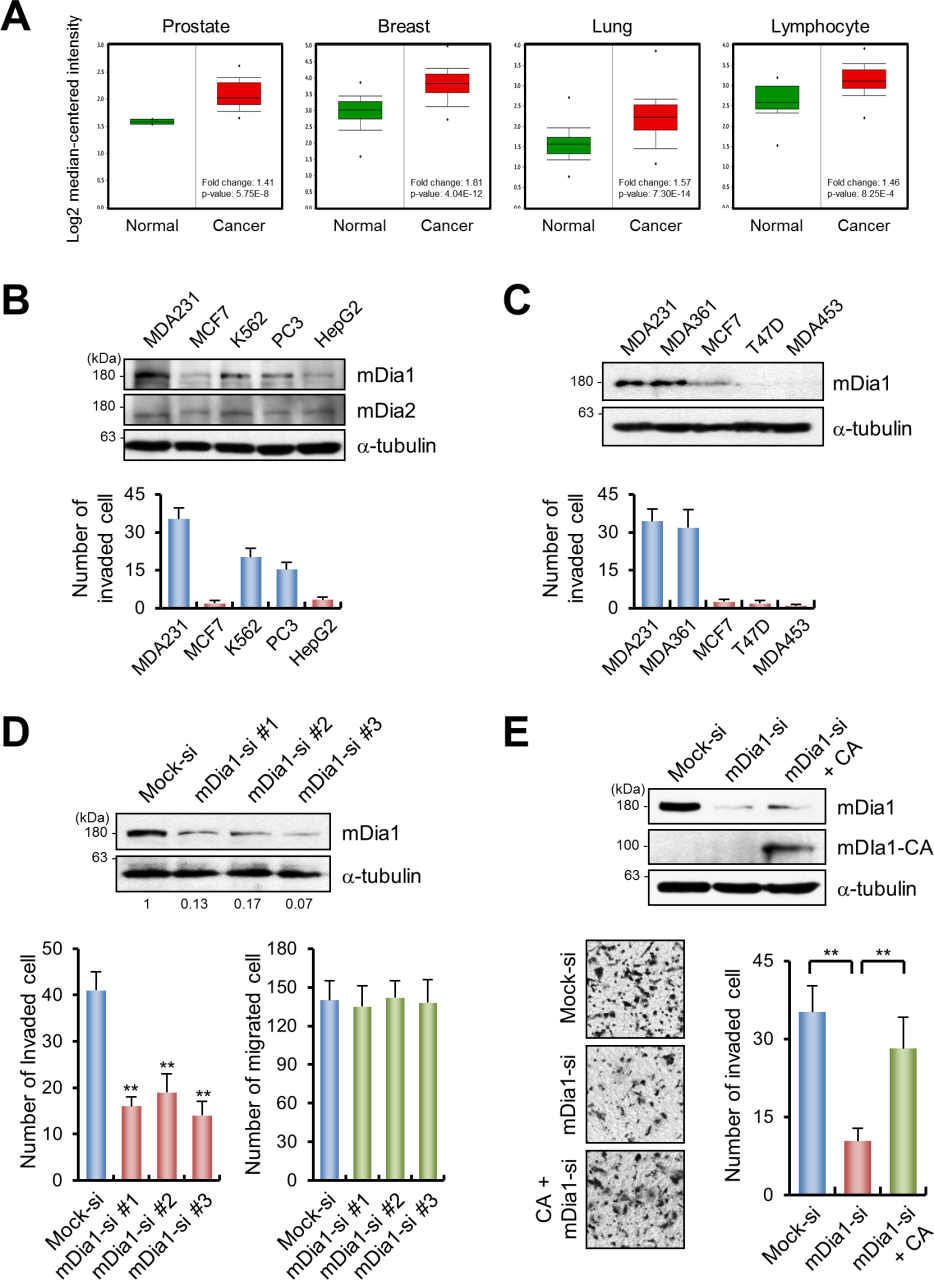
mDia1 stimulates breast cancer invasion **A.** Analysis of mDia1 gene expression in prostate, breast, lung and lymphocyte using the Oncomine database. **B, C.** Western blotting and transwell migration assays showed that MDA-MB-231 cells are highly motile and invasive. Expression of mDia1 was higher in invasive breast cancer cells (MDA-MB-231 and MDA-MB-361) than in non-invasive cancer cells (MCF7, T47D, and MDA-MB-453). **D.** Western blot analysis demonstrating the downregulation of mDia1 in MDA-MB-231 cells transfected with siRNA against mDia1 (mDia1-si #1, #2, and #3) compared with mock-transfected cells (Mock-si). Numbers under the blot indicate relative levels of mDia1 expression in the samples. siRNA-mediated silencing of mDia1 led to a significant decrease in invasive capability, but not in motility. **E.** Western blot analysis comparing the expression of mDia1 among MDA-MB-231 cells transfected with mock siRNA (Mock-si), mDia1 siRNA (mDia1-si), or the constitutively active form of mDia1 (mDia1-CA). Knockdown of mDia1 resulted in decreased invasiveness, which was rescued by the forced expression of mDia1-CA. Images were obtained by light microscopy. **p < 0.01.

According to the cancer profiling database Oncomine (http://www.oncomine.org), transcription of mDia1 is elevated in several cancers, including breast cancer (Figure 1A). We investigated the correlation between mDia1 protein expression levels and invasive potential, in several invasive and non-invasive cancer cell lines. As shown in Figure 1B, cells expressing mDia1 exhibited invasive capacity, whereas mDia2 was not correlated with cancer invasion due to its weak expression. To confirm the relationship between mDia1 expression and the invasive activity of malignant cancer cells, we examined mDia1 expression levels in several malignant breast cancer cell lines (Figure 1C). Western blot analysis showed that aggressive cancer cell lines, such as MDA-MB-231 and MDA-MB-361, expressed higher levels of mDia1 protein than non-invasive cell lines (MCF-7, T47D, and MDA-MB-453), indicating that the expression of mDia1 was closely correlated with breast cancer cell invasion.

To examine the role of mDia1 in cancer invasion, mDia1 expression in MDA-MB-231 cells was silenced using three different short interfering RNAs (siRNAs), and the cells were then subjected to migration and invasion analyses. Western blotting showed that mDia1 levels were significantly reduced by all the tested siRNAs as compared to the mock-transfected cells, without changing tubulin levels (Figure 1D). Invasion of mDia1-silenced cells on collagen-coated transwells was significantly reduced as compared to that of the mock-transfected cells, with no difference in cell migration ability observed on uncoated transwell inserts (Figure 1D). Decreased invasiveness of mDia1-silenced cells was significantly restored to levels similar to those of controls by overexpression of the constitutively active (CA) form of mDia1 (mDia1-CA), indicating that mDia1 is directly involved in cancer cell invasion (Figure 1E).

### mDia1-mediated cancer invasion is dependent on MT1-MMP localization

**Figure 2:**
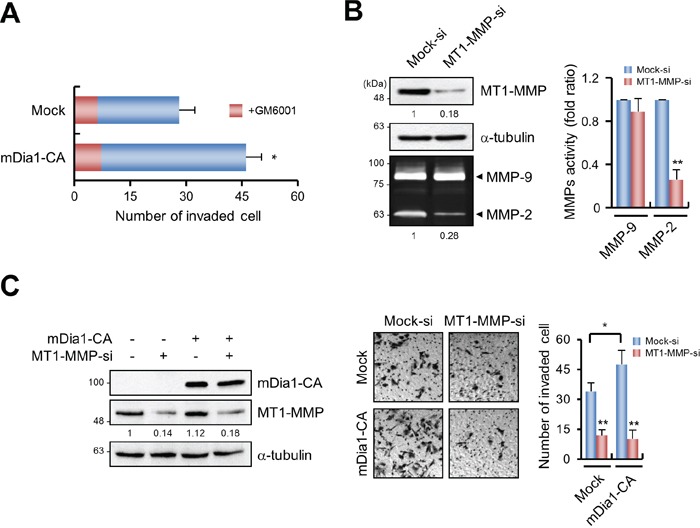
mDia1 stimulates invasion through MT1-MMP **A.** Invasion of MDA-MB-231 cells transfected with mock and mDia1-CA plasmid was measured using transwells. GM6001 (5 μM) was added to both cells to prevent MMP-dependent invasion. Invasiveness induced by overexpression of mDia1-CA was significantly decreased in the presence of 5 μM GM6001. **B.** Western blot analysis demonstrating the expression level of MT1-MMP in MDA-MB-231 cells transfected with mock siRNA (Mock-si) or MT1-MMP siRNA (MT1-MMP-si). Total cell lysates were subjected to Western blot analysis using an antibody against MT1-MMP. Expression of MT1-MMP was markedly decreased following siRNA silencing, resulting in reduced MMP-2 activity. Numbers under the blot and zymography gels indicate relative levels of MT1-MMP and MMP-2 activity in the samples, respectively. **C.** Western blot analysis demonstrating mDia1-CA and MT1-MMP expression levels in MDA-MB-231 cells transfected with mDia1-CA plasmid and MT1-MMP siRNA. Numbers under the blot indicate relative levels of MT1-MMP in the samples. Cells were subjected to transwell invasion assay. Images were obtained by light microscopy. *p < 0.05, ** p < 0.01.

The invasive ability of various cancers is strongly associated with MMP activity [3, 18]. Therefore, we tested whether mDia1-mediated invasion is dependent on MMP activity. Forced expression of mDia1-CA significantly induced invasiveness of MDA-MB-231 cells (Figure 2A). After treatment with GM6001, a general MMP inhibitor, invasive activity was reduced by similar levels in mock-transfected cells and those transfected with mDia1-CA (Figure 2A), indicating the involvement of MMP activity in mDia1-mediated cancer cell invasion.

MT1-MMP is a major MMP involved in the invasion of MDA-MB-231 cells [19]. Therefore, we tested whether mDia1-induced invasion of MDA-MB-231 cells requires MT1-MMP activity. Transfection of MT1-MMP-specific siRNAs into MDA-MB-231 cells resulted in considerably decreased levels of MT1-MMP protein expression (Figure 2B). MMP-2 is known to be activated through a MT1-MMP-dependent process [20, 21]; therefore, we assessed MMP-2 activity in MT1-MMP silenced cells. As shown in Figure 2B, MMP-2 activity was significantly reduced in MT1-MMP-silenced cells, while MMP-9 activity was unaffected. In addition, the enhanced invasiveness conferred by mDia1-CA overexpression was decreased by MT1-MMP siRNAs, to levels similar to those in mock-transfected cells (Figure 2C).

**Figure 3:**
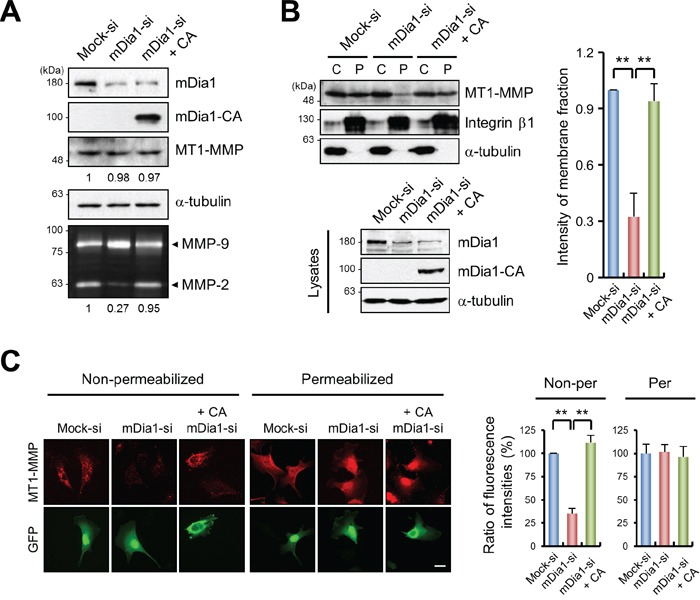
mDia1 regulates membrane localization of MT1-MMP **A.** Western blot analysis demonstrating MT1-MMP expression levels in MDA-MB-231 cells transfected with mock siRNA (Mock-si), mDia1 siRNA (mDia1-si), or mDia1 siRNA plus mDia1-CA plasmid (mDia1-si + CA). Cell lysates were subjected to Western blot analysis using an antibody against MT1-MMP. MMP-9 and MMP-2 activities were determined by gelatin zymography. Numbers under the blot and zymography gels indicate relative levels of MT1-MMP and activity of MMP-2 in the samples, respectively. **B.** Membrane fractionation assay was performed using MDA-MB-231 cells transfected with mock siRNA (Mock-si), mDia1 siRNA (mDia1-si), or mDia1 siRNA plus mDia1-CA plasmid (mDia1-si + CA). Cell lysates were subjected to Western blot analysis using antibodies against mDia1, Integrin β1, and α-tubulin. Integrin β1 and α-tubulin were used as positive controls for the plasma membrane and the cytosolic fractions, respectively [54, 55]. C, cytosol; P, plasma membrane. **C.** Cells transfected with mock siRNA (Mock-si), mDia1 siRNA (mDia1-si), or mDia1 siRNA plus mDia1-CA plasmid (mDia1-si + CA) were fixed, and then stained with an antibody against MT1-MMP followed by a secondary Cy3-conjugated antibody to label endogenous MT1-MMP on the cell surface or in whole cells. Scale bar, 20 μm. **p < 0.01.

To determine the mechanistic aspects of how mDia1 controls MT1-MMP to induce breast cancer invasion, we first examined whether knockdown or overexpression of mDia1 regulates MT1-MMP expression, since it was reported that silencing of mDia1 also suppressed the expression of myogenic regulatory factor proteins such as MyoD [22]. Western blot analysis showed that MT1-MMP levels were not changed upon mDia1 silencing or when mDia1-CA was re-introduced (Figure 3A). However, MMP-2 activity was markedly reduced as a consequence of mDia1 knockdown, which was restored to control levels after transfection with mDia1-CA (Figure 3A). MT1-MMP localizes to the plasma membrane to induce MMP-2 activation [20], and that intracellular trafficking of subcellular organelles may be regulated by mDia1 [23, 24]; therefore, we hypothesized that mDia1 could be involved in the localization of MT1-MMP to the plasma membrane to induce cancer cell invasion. To test this hypothesis, we first performed membrane fractionation assays. In cells in which mDia1 was silenced, translocation of MT1-MMP to the plasma membrane was significantly reduced. In addition, overexpression of mDia1-CA in mDia1-silenced cells restored the localization of MT1-MMP to the plasma membrane, confirming that MT1-MMP localization to the plasma membrane was dependent on mDia1 (Figure 3B). Immunocytochemistry and flow cytometry analysis for total or cell surface localized endogenous MT1-MMP showed that mDia1 silencing completely inhibited the localization of MT1-MMP to the plasma membrane. Conversely, ectopic expression of mDia1-CA in the mDia1-silenced cells restored the cell-surface localization of MT1-MMP (Figure 3C and Supplementary Figure S1). These findings indicate that knockdown of mDia1 resulted in reduced abundance of cell surface localized endogenous MT1-MMP, but not the level of MT1-MMP expression in whole cells.

### mDia1 regulates MT1-MMP localization on the cell surface

**Figure 4:**
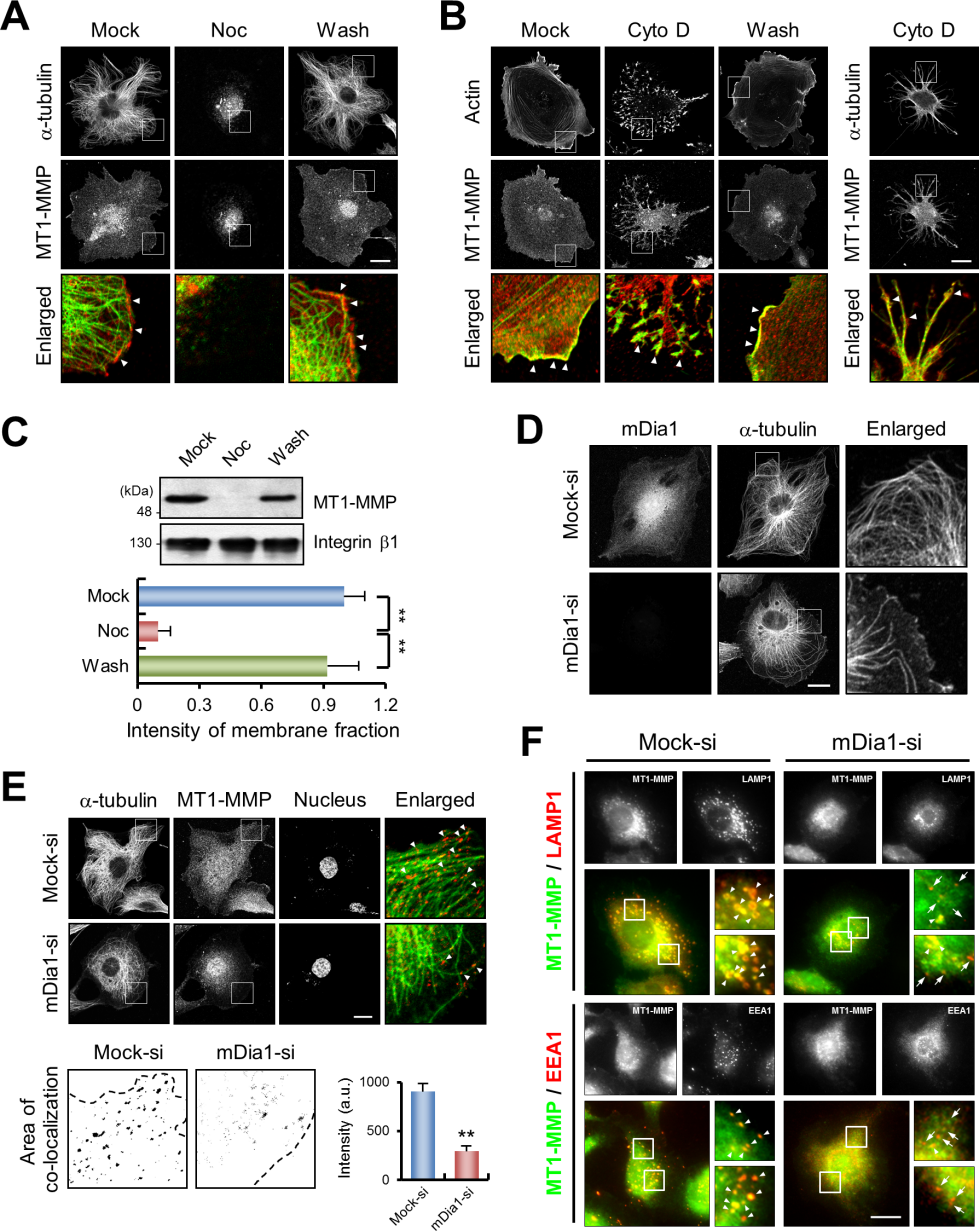
mDia1 affects MT1-MMP localization by modulating microtubule dynamics **A.** Immunofluorescence labeling confocal microscopy for α-tubulin (green) and MT1-MMP (red) in nocodazole-treated MDA-MB-231 cells. Cells were treated with 5 μM nocodazole for 1 h. Mock, mock-treated cells; Noc, nocodazole; Wash, nocodazole was washed out of cells. Arrowheads indicate co-localization of α-tubulin and MT1-MMP. Scale bar, 20 μm. **B.** Similar experiments were performed as in (A.) but by using cells treated with cytochalasin D. Cells were stained with Alexa Fluor^®^ 488 phalloidin for actin (green) and antibody against MT1-MMP (red) (left). One of the same samples was also stained with antibodies against α-tubulin (green) and MT1-MMP (red) (right). Mock, mock-treated cells; Cyto D, cytochalasin D; Wash, cytochalasin D was washed out of cells. Arrowheads indicate co-localization of actin and MT1-MMP (*left*) and of α-tubulin and MT1-MMP (*right*). Scale bar, 20 μm. **C.** Membrane fractionation assay was performed using nocodazole-treated MDA-MB-231 cells. Cell lysates were subjected to Western blot analysis with antibodies against MT1-MMP and integrin β1. Integrin β1 was used as a positive control for the plasma membrane fraction. **D.** Immunofluorescence labeling confocal microscopy for α-tubulin and mDia1 in MDA-MB-231 cells transfected with mock siRNA (Mock-si) and mDia1 siRNA (mDia1-si). Scale bar, 20 μm. **E.** Nocodazole-treated mock siRNA-transfected (Mock-si) and mDia1-silenced cells (mDia1-si) were washed to re-polymerize microtubules, then fixed and stained with antibodies against α-tubulin (green), MT1-MMP (red), and 4′,6-diamidino-2-phenylindole (DAPI). Arrowheads indicate co-localization of MT1-MMP and α-tubulin (*upper*). Scale bar, 20 μm. More detailed area of co-localization of MT1-MMP and α-tubulin in cell peripheral region is represented (*lower*), and their intensity was quantified. Co-localization of MT1-MMP and α-tubulin in mDia1-silenced cells was significantly decreased. Dashed line indicates cell outline. **F.** MDA-MB-231 cells transfected mock siRNA (Mock-si) and mDia1 siRNA (mDia1-si) were fixed and stained with antibodies against MT1-MMP (green) and LAMP1 (red) or EEA1 (red), respectively, and analyzed by TIRFM. Insets are higher magnification views of boxed areas. Arrowheads indicate co-localization of MT1-MMP and LAMP1 or EEA1, and arrows indicate lesser or non-co-localization of MT1-MMP and LAMP1 or EEA1. Scale bar, 20 μm. **p < 0.01.

An intact network of α-tubulin is necessary for the localization of MT1-MMP to the plasma membrane [4]. As shown in Figure 4A, disruption of microtubule structure using nocodazole resulted in abnormal intracellular distribution of MT1-MMP around the nucleus. In contrast, washing out nocodazole to induce re-polymerization of microtubules restored the normal intracellular distribution of MT1-MMP, which was similar to that of the microtubule network (Figure 4A). However, disruption of the actin cytoskeleton by treatment with cytochalasin D did not change the normal cellular distribution of MT1-MMP proximal to the plasma membrane (Figure 4B), which indicates that the transport of MT1-MMP is dependent on microtubules, and not on actin filaments. Furthermore, membrane fractionation assays with nocodazole treated cells confirmed that disruption of the microtubule structure decreased the localization of MT1-MMP to the plasma membrane (Figure 4C), suggesting that proper microtubule formation is critical for MT1-MMP localization to the plasma membrane.

As mDia1 is known to regulate microtubule polymerization [7], our results also showed that mDia1 silencing prevented microtubule outgrowth (Figure 4D) consistent with a previous report [25]. Therefore, we examined the distribution of MT1-MMP in mDia1-silenced cells. As shown in Figure 4E, a large proportion of MT1-MMP-positive vesicles were dispersed around the nucleus, resulting in significantly decreased peripheral co-localization between MT1-MMP and microtubules in mDia1-silenced cells (Figure 4E). Since vesicle-associated membrane protein 7 (VAMP7) and Rab7 respond to regulate the fusion of MT1-MMP containing endosomes with the plasma membrane [26, 27], we further investigated whether mDia1-silencing leads to decreased interaction between MT1-MMP and early endosome (EEA1) or late endosome/lysosome (LAMP1). As observed by total internal reflection fluorescence (TIRF) microscopy, we found that cell surface MT1-MMP and LAMP1 were strongly co-localized, which was decreased upon knockdown of mDia1, and similar results were observed between MT1-MMP and EEA1 (Figure 4F). Since the trafficking of these endosomes is dependent on microtubules [27], and knockdown of mDia1 decreases microtubule outgrowth, it could be possible that decrease in cell surface localization of MT1-MMP might be triggered by defects in the trafficking of these endosomes upon mDia1 knockdown. Taken together, these results suggest the possibility that silencing mDia1 might inhibit the interaction between MT1-MMP and microtubules, and thereby decrease cell surface MT1-MMP localization.

### mDia1 is required for interaction between MT1-MMP and microtubules

**Figure 5:**
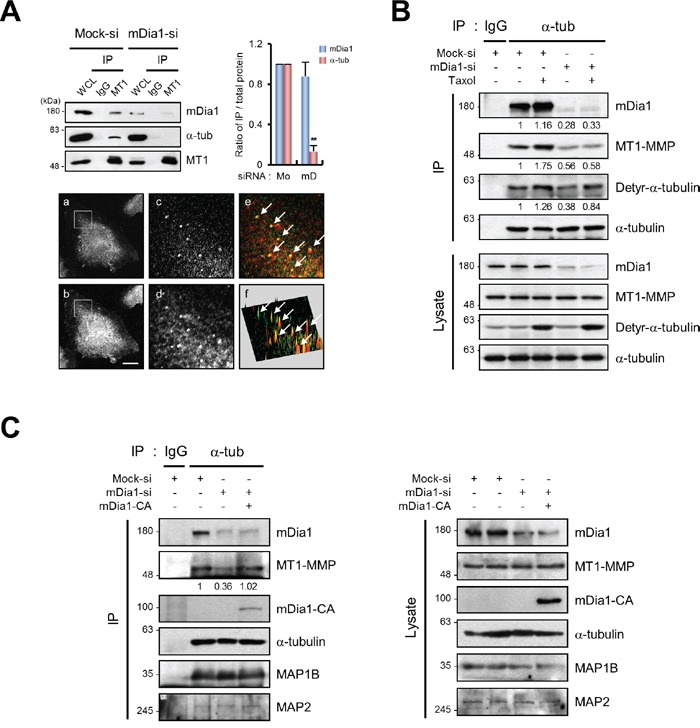
mDia1 regulates interaction between MT1-MMP and microtubules **A.** Immunoprecipitation (IP) and Western blot analysis demonstrating reduction in MT1-MMP binding to microtubules in MDA-MB-231 cells transfected with siRNA for mDia1 (mDia1-si) compared with that in mock transfected cells (Mock-si). Cell lysates were subjected to IP with an antibody against MT1-MMP, followed by Western blot analysis with antibodies against mDia1, α-tubulin, and MT1-MMP. Relative ratio of IP over total protein expression was quantified and presented. MT1, MT1-MMP; Mo, Mock; mD, mDia1. Immunofluorescence was used to visualize mDia1 (red) and MT1-MMP (green). (a) mDia1 immunofluorescence; (b) MT1-MMP immunofluorescence; (c) enlarged inset area from (a); (d) enlarged inset area from (b); (e) merged image of (c) and (d) showing areas of co-localization; (f) reconstruction of co-localized fluorescence peaks. The arrows indicate co-localization of MT1-MMP and mDia1. Scale bar, 20 μm. **B.** IP and Western blot analysis demonstrating a decrease in the amount of interaction between MT1-MMP and α-tubulin in MDA-MB-231 cells transfected with siRNA for mDia1 (mDia1-si) compared with mock siRNA-transfected cells (Mock-si). Cell lysates were subjected to IP using an antibody against α-tubulin, followed by Western blot analysis using antibodies against mDia1, MT1-MMP, detyrosinated-α-tubulin, and α-tubulin. Detyrosinated-α-tubulin was used as an internal control to demonstrate that mDia1 specifically regulates MT1-MMP binding to microtubules. Numbers under the blot indicate relative levels of IP over total protein expression in the samples. **C.** IP and Western blot analysis showing a decrease in the amount of interaction between MT1-MMP and α-tubulin in MDA-MB-231 cells transfected with siRNA for mDia1 (mDia1-si), which was markedly rescued by forced expression of mDia1-CA. Cell lysates were subjected to IP using an antibody against α-tubulin, followed by Western blot analysis with antibodies against mDia1, MT1-MMP, GFP, α-tubulin, microtubule-associated protein (MAP) 1B, and MAP2. MAP1B and MAP2 were used to confirm the specificity of interactions among mDia1, MT1-MMP, and α-tubulin. Numbers under the blot indicate relative levels of IP over total protein expression in the samples. **p < 0.01.

MT1-MMP is known to interact with α-tubulin [28]. Therefore, we performed immunoprecipitation (IP) assays to investigate whether mDia1 influences this interaction. Figure 5A shows that MT1-MMP was bound to mDia1 and α-tubulin in mock-transfected cells (Figure 5A). Immunofluorescence images also confirmed that mDia1 (Figure 5Aa and 5Ac) and MT1-MMP (Figure 5Ab and 5Ad) were colocalized (Figure 5Ae and 5Af). However, mDia1 silencing markedly diminished the amount of interaction between MT1-MMP and α-tubulin (Figure 5A), indicating that the interaction of MT1-MMP with microtubules is decreased by the silencing of mDia1.

Since increased microtubule stability is known to enhance the binding affinity of microtubules for interacting proteins [29, 30], we examined whether decreased amount of interaction between MT1-MMP and microtubules in mDia1-silenced cells could be due to decrease in microtubule stability [31]. The results presented in Figure 5B show that the interaction between MT1-MMP and α-tubulin was significantly increased following Taxol treatment in mock-transfected cells. However, the binding of MT1-MMP to α-tubulin was not restored in mDia1-silenced cells when microtubules were stabilized by Taxol treatment (Figure 5B). These results indicate that mDia1 mediates the binding of MT1-MMP to microtubules directly but not through microtubule stabilization. In addition, we observed that reintroduction of mDia1-CA into mDia1-silenced cells restored the decreased MT1-MMP binding to microtubules caused by knockdown of mDia1 (Figure 5C). Interestingly, the expression of other microtubule-associated proteins (MAPs) including MAP1B and MAP2 was not significantly changed by mDia1 silencing or by the forced expression of mDia1-CA, indicating that this interaction is specific to mDia1 and MT1-MMP (Figure 5C). Thus, the results clearly indicate that the mDia1 for intracellular trafficking of MT1-MMP may be required for adequate binding of MT1-MMP to α-tubulin.

### mDia1 is required for breast cancer invasion in a 3D environment

**Figure 6:**
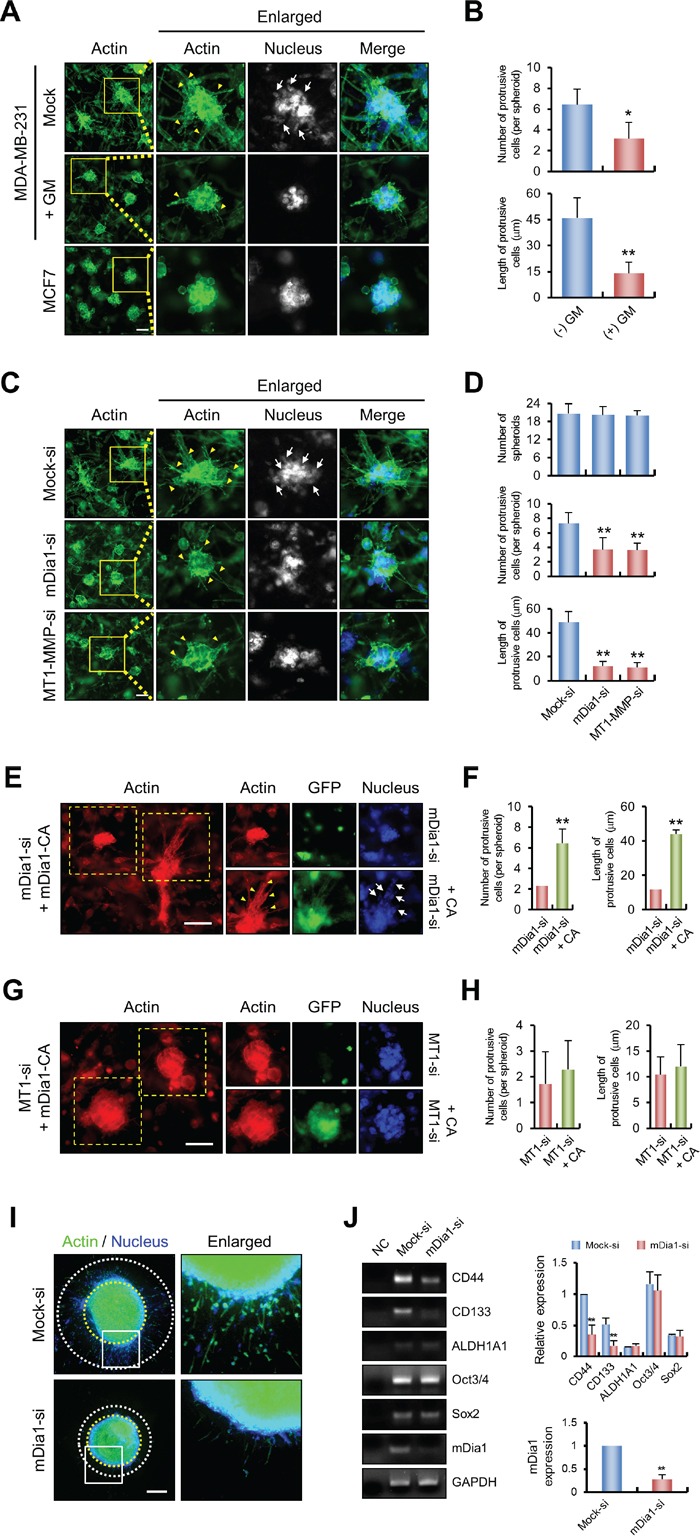
mDia1 is required for breast cancer invasion in 3D environments **A, B.** Immunofluorescence labeling demonstrating protrusive phenotypes of MDA-MB-231 cells treated with or without GM6001 (5 μM) and of MCF7 cells. MDA-MB-231 and MCF7 cells were mixed with collagen (2 mg/ml) and Matrigel^®^ (1 mg/ml), then allowed to polymerize for 1 h. 3D matrices were incubated for 6 days to allow spheroid formation. Samples were fixed and stained with phalloidin and DAPI to visualize cellular actin and nuclei, respectively. The histogram shows the number of protrusive cells per spheroid and the lengths of protrusive cells in untreated MDA-MB-231 cells compared with those of cells treated with GM6001. **C, D.** Similar experiments were performed using MDA-MB-231 cells transfected with mock siRNA (Mock-si), mDia1 siRNA (mDia1-si), and MT1-MMP siRNA (MT1-MMP-si). The histogram shows the number of spheroids in each matrix, the number of protrusive cells per spheroid, and the lengths of protrusive cells in MDA-MB-231 cells transfected with siRNA against mDia1 (mDia1-si) and MT1-MMP (MT1-MMP-si) compared with those of mock transfected cells (Mock-si). Arrowheads and arrows indicate the protrusive phenotype and cells, respectively. Scale bar, 50 μm. *p < 0.05, ** *p* < 0.01 **E, F.** Similar experiments were performed using MDA-MB-231 cells transfected with mDia1 siRNA (mDia1-si) and mDia1 siRNA plus mDia1-CA plasmid (mDia1-si + CA). As shown by the histogram, the number of protrusive cells per spheroid and the lengths of protrusive cells were decreased in mDia1 siRNA transfected cells and significantly rescued by overexpression of mDia1-CA. Scale bar, 50 μm. **G, H.** Similar experiments were performed using MDA-MB-231 cells transfected with MT1-MMP siRNA (MT1-si) and MT1-MMP siRNA plus mDia1-CA plasmid (MT1-si + CA). As shown by the histogram, the number of protrusive cells per spheroid and the lengths of protrusive cells were decreased in MT1-MMP siRNA transfected cells, which was not markedly restored by overexpression of mDia1-CA. Scale bar, 50 μm. **I, J.** MDA-MB-231 cells transfected with mock siRNA (Mock-si) and mDia1 siRNA (mDia1-si) were incubated for 2 days to form spheroids as described in *Materials and Methods*. Collagen embedded spheroids were further incubated for 4 days to allow cell invasion. Samples were fixed and stained with phalloidin and DAPI to visualize cellular actin and nuclei, respectively. At the end of incubation, total RNA of the samples were extracted and used for detection of CD44, CD133, ALDH1A1, Oct3/4, and Sox2 mRNA expression by RT-PCR (*left*) and qRT-PCR (*right*). Scale bar, 400 μm. *p < 0.05, ** *p* < 0.01.

We next set up an experiment to determine the effect of altered mDia1 expression on MDA-MB-231 cell invasion using a 3D cell culture system. MDA-MB-231 cells cultured in 3D matrices composed of collagen and Matrigel^®^ exhibited a protrusive phenotype, as previously reported [32]. To confirm whether the protrusive phenotype reflected invasive activity, cells were treated with the general MMP inhibitor GM6001 followed by analysis of their morphometric characteristics. The protrusive phenotype was markedly decreased by treatment with GM6001, and the length of the protrusions was considerably reduced (Figure 6A and 6B). This finding was similar to our observations of spheroid generation by non-invasive MCF-7 cells (Figure 6A). Thus, we concluded that this protrusive phenotype was representative of invasive cells in 3D matrices. Based on these results, we investigated whether mDia1 knockdown suppressed the protrusive phenotype of invasive breast cancer cells. Inhibition of mDia1 expression resulted in a significant reduction in the number of protrusive cells in each spheroid, while the number of spheroids generated by mDia1-silenced cells was not significantly altered in 3D matrices (Figure 6C and 6D). Knockdown of MT1-MMP expression also decreased the number of protrusive cells in the spheroid (Figure 6C and 6D). Based on our observation that mDia1-CA rescued decreased cell invasion (Figure 1 and 2), we presumed that overexpression of mDia1-CA in mDia1-silenced cells would result in restoration of the protrusive phenotype of spheroids. As shown in Figure 6E and F, forced expression of mDia1-CA led to recovery of the protrusive phenotype in spheroids as measured by the number and length of protrusive cells, compared with that of mDia1-silenced cells (Figure 6E and 6F). We performed similar experiments with MT1-MMP silenced cells, and in agreement with the results obtained in Figure 2C, overexpression of mDia1-CA did not restore the protrusive phenotype in MT1-MMP-silenced cells (Figure 6G and 6H).

As spheroids formed in soft 3D conditions may have some stem cell characteristics [33], and since cancer stem cell (CSC) gene expression is important for cancer progression and self-renewal [34], we further investigated whether knockdown of mDia1 in 3D spheroids leads to changes in gene expression of CSC markers. We generated spheroids using multicellular spheroid culture, which is one of the most classical and widely used methods to investigate 3D spheroids [35, 36], with minor modification. Before the experiments, we confirmed that our modified multicellular spheroid culture system works properly by using the invasion assay. As shown in Figure 6I, mock-transfected cells invaded into 3D collagen matrices, whereas invasion of mDia1-silenced cells was reduced. Under this condition, cells were used for RNA isolation, and a panel of CSC and self-renewal markers—CD44, CD133, ALDH1A1, Oct3/4, and Sox2—were examined by reverse transcription-polymerase chain reaction (RT-PCR). The expression of ALDH1A1, Oct3/4 and Sox2 was not markedly changed by knockdown of mDia1, whereas the expression of CD44 and CD133 was largely decreased (Figure 6J, left), and these results were further confirmed with quantitative RT-PCR (Figure 6J, right). Since CD44 and CD133 are involved in cancer progression [37, 38], and CD44 regulates cancer invasion through MT1-MMP [39], these results suggested that decreased expression of these CSC markers, especially CD44 and CD133, by knockdown of mDia1 may be involved in decreased cell invasion in the 3D environment. Taken together with above findings, these results suggest that interfering with mDia1 expression *in vivo* may inhibit breast cancer invasion, at least to some extent.

## DISCUSSION

Cancer cell invasion and metastasis is accompanied by extensive morphological changes; therefore, the rearrangement of cytoskeletal proteins and its related signals has been studied extensively [1–4]. mDia1 is well known as an actin dynamics regulator during the process of cancer cell invasion, although it has the ability to bind to microtubules and regulate microtubule dynamics [5]. In particular, diaphanous-related proteins, including mDia1 are components of the invadopodia, which is an actin-based specialized structure in invasive cancer cells that can degrade ECM [14, 26]. MT1-MMP is known to be an indispensable factor for cancer cell invasion [13, 19]. Although intracellular trafficking of MT1-MMP to localize to target structures such as the invadopodia is critical for cancer cell invasion, it is not well established how MT1-MMP localizes to its specialized target. In this regard, our results demonstrated that mDia1 functions as a bridge between MT1-MMP and microtubule track to deliver MT1-MMP to its proper localization site. In addition, mDia1 also can regulate microtubule dynamics, which is necessary for intracellular trafficking of MT1-MMP via interaction with endosomes.

MT1-MMP is endocytosed from the plasma membrane and recycled to the cell surface by early and late endosomal structures. When MT1-MMP is internalized from the cell surface, it is complexed with EEA1 and is colocalized with Rab4, a marker for recycling endosomes, during recycling to the cell surface [40]. In addition, trafficking and recycling of MT1-MMP is dependent upon Rab7 and VAMP7 [41]. Furthermore, MT1-MMP was found to be colocalized with LAMP1 [26, 40]. Consistent with the above reports, our results showed that MT1-MMP and EEA1 or LAMP1 are both colocalized on the cell surface (Figure 4F), which was reduced by knockdown of mDia1. Since endosomes can move along microtubules, especially through motor proteins such as kinesin and dynein [27], these results could be triggered by decreased microtubule stabilization upon knockdown of mDia1. However, reduced interaction between MT1-MMP and microtubules was due to decreased expression of mDia1, and not microtubule stability (Figure 5), suggesting that mDia1 is involved in endosomal trafficking for MT1-MMP transport. Although mDia proteins were detected in endosomes, their functional significance remains unclear [17], and therefore, their detailed mechanism needs to be further elucidated.

Since mDia1 has previously been identified as a regulator of actin filaments, most prior reports have suggested that mDia1 plays a role in cell invasion through actin assembly [12, 14, 42]. It was also reported that the actin-binding protein, cortactin, modulates the secretion and membrane expression of invadopodia-associated MMPs including MMP-2, MMP-9, and MT1-MMP [43]. Since knockdown of mDia1 expression also reduces expression of cortactin [44], it could not be excluded that mDia1 may modulate MT1-MMP localization at the invadopodia via regulation of cortactin expression. It was reported that disassembly of microtubules by nocodazole treatment inhibits invadopodia biogenesis, leading to decrease ECM degradation, even though preformed invadopodia are not affected [45]. It still remains unclear whether microtubules are essential for direct mechanical support of invadopodia [46]. However, disruption of microtubules decreases MMP trafficking, and therefore, microtubules might be an essential factor in the polarized transport of invadopodia-associated proteins including MT1-MMP [45]. We showed that the localization of MT1-MMP at the plasma membrane is critical for breast cancer invasion and occurred through proper microtubule formation and elongation via mDia1, and not by actin filament formation (Figure 4). The disruption of microtubule formation and elongation, by both nocodazole treatment and mDia1 silencing resulted in decreased abundance of MT1-MMP in the plasma membrane. mDia1 is essential for binding of MT1-MMP to α-tubulin; thus, in addition to cortactin and actin enriched invadopodia, mDia1 may have functions in breast cancer invasion through mDia1-mediated microtubule regulated signaling.

Over the last decade, 3D cell culture systems have been used to mimic *in vivo* conditions. Although it is difficult to reproduce the exact conditions of a living organism in such systems, they are invaluable for investigating cell-cell and cell-matrix interactions [47]. When breast cancer cells invade or metastasize to another organ, they encounter the basement membrane, which is primarily formed of collagen, the most abundant protein in the human body [48]. To simulate the invasion of tumor cells through the basement membrane, we set out to generate 3D matrices composed of collagen and Matrigel^®^ in an effort to mimic the complex extracellular environment found in various tissues [49, 50]. Knockdown of mDia1 led to a significant decrease in the invasive phenotype, which was restored by forced expression of mDia1-CA (Figure 6). Silencing of MT1-MMP produced a similarly less invasive phenotype in mDia1-silenced cells, suggesting that mDia1 could stimulate breast cancer invasive potential under *in vivo*-like conditions via MT1-MMP. Reduced experimental time and easy reproducibility for the observation of cellular invasive activity under *in vivo*-like 3D conditions, are the strong points of our 3D cell culture system, which can be helpful for the study of tumorigenesis modulation by drugs or by inhibition of specific proteins. In addition, we found that the expression of CSC marker genes such as CD44 and CD133 in the 3D environment was decreased by the knockdown of mDia1 (Figure 6J). It is well known that alterations in formin function and expression are directly involved in diseases including cancer [51, 52], and that CSCs are regarded as the basis for cancer initiation as well as cancer progression and metastasis [34]. Due to self-renewal characteristics of cancer cells, patients with cancer usually suffer from recurrence and metastasis because current therapies cannot eliminate CSCs, indicating that elimination of CSCs is extremely important for treating malignant diseases [34, 53]. Although the detailed mechanism of how formin proteins participate in cancers has not been fully elucidated [23], downregulation of mDia1 decreased CSC marker expression, indicating the existence of a close relationship between mDia1 expression and CSC development during cancer progression. In this regard, the results of this study suggested that mDia1 could be a useful therapeutic target for breast cancer treatment.

## MATERIALS AND METHODS

### Reagents

TransFectin™ Lipid Reagent was obtained from Bio-Rad (Hercules, CA, USA). Oligofectamine™ Transfection Reagent was purchased from Invitrogen (Carlsbad, CA, USA). Fetal bovine serum (FBS) was purchased from Biowest (Nuaillé, France). Antibodies against mDia1 (sc-10888), GFP (sc-9996), MT1-MMP (sc-12367 and sc-30074), EEA1 (sc-6415), and MAP1B (sc-8971) were from Santa Cruz Biotechnology (Dallas, TX, USA). Antibodies against α-tubulin (T9026) and detyrosinated-α-tubulin (AB3201) were obtained from Sigma-Aldrich (St. Louis, MO, USA) and Millipore (Billerica, MA, USA), respectively. MAP-2 antibody (MAP3418) and LAMP1 antibody (ab25630) were purchased from Chemicon (Billerica, MA, USA) and Abcam (Cambridge, MA, USA), respectively. Protein A-agarose beads were purchased from GenDEPOT (Barker, TX, USA). Rat tail type I collagen and Matrigel^®^ were purchased from BD Biosciences (Franklin Lakes, NJ, USA).

### Cell culture

The MDA-MB-231 and MCF-7 cell lines were purchased from the Korea Cell Line Bank (Seoul, Korea). The MDA-MB-361, MDA-MB-453, and T47D cell lines were obtained from Professor Dongeun Park (Seoul National University, Korea), while K562, HepG2, and PC3 cell lines were from Professor Sangbeom Seo (Chung-Ang University, Korea). All cell lines were cultured in RPMI 1640 medium supplemented with 10% (v/v) FBS, 100 units/ml penicillin, and 100 μg/ml streptomycin (Welgene, Seoul, Korea). Cultures were incubated at 37°C with 5% CO_2_.

### Plasmids and transfection

The construct for mDia1-CA was subcloned into pEGFP-N1. All cloned sequences were verified by DNA sequencing. Transient transfection was performed by using TransFectin™ Lipid Reagent or a Neon^®^ electroporation device (Invitrogen), according to the manufacturer's instructions.

### siRNA transfection

We purchased siRNAs specific for mDia1 #1 (5′-AAAGGCAGAGCCACACUUCCU-3′), mDia1 #2 (5′-GCUGGUCAGAGCCAUGGAU-3′), mDia1 #3 (5′-GCAUGAGAUCAUUCGCUGC-3′), and MT1-MMP (5′-GAAGCCUGGCUACAGCAAUAU-3′), along with a control siRNA (5′-AUUGUAUGCGAUCGCAGAC-3′) from Genolution (Seoul, Korea). Cells were transfected with the appropriate quantity of siRNA using Oligofectamine™ reagent according to the manufacturer's recommended protocol.

### Co-immunoprecipitation and western blotting

MDA-MB-231 cells were transfected with the indicated siRNA or plasmid for 48 h. Cell lysates were incubated with antibodies against MT1-MMP or α-tubulin, and further incubated with Protein A-agarose beads. Solubilized bead-bound materials were subjected to sodium dodecyl sulfate polyacrylamide gel electrophoresis (SDS-PAGE) and then transferred to polyvinylidene difluoride membranes (Millipore). Membranes were blocked, washed, and incubated with primary and secondary antibodies as indicated. Signals were developed using enhanced chemiluminescence reagent and band density was measured using a Quantity One^®^ system (Bio-Rad).

### Immunofluorescence microscopy

Cell preparations were fixed for 15 min with 3.7% (w/v) paraformaldehyde in phosphate-buffered saline (PBS), then blocked with 2% (w/v) bovine serum albumin (BSA) in PBS for 1 h. Samples were permeabilized for 10 min with 0.5% (v/v) Triton™ X-100 in PBS. Cells were then incubated with a primary antibody for 1 h, followed by incubation with the appropriate secondary antibody for 1 h at room temperature. Samples were analyzed using an Eclipse 80i fluorescence microscope (Nikon, Tokyo, Japan) or an LSM 700 confocal microscope (Carl Zeiss, Oberkochen, Germany). Images were acquired using a digital camera (DS-Qi1Mc, Nikon) and NIS-Elements image analysis software (Nikon). TIRF images were acquired using an inverted Olympus IX70 microscope (Olympus, Melville, NY, USA) equipped with a 60× Apochromat N objective (NA 1.49). FITC and Cy3 were excited with 488- and 553-nm lasers, respectively. Images were acquired using a charge-coupled device (CCD) camera (Retiga EXi; QImaging) and MetaMorph software (Molecular Devices). Image processing was carried out using Photoshop 11.0 (Adobe, San Jose, CA, USA).

### Separation of plasma membrane and cytosolic fractions

Cells were washed and then suspended in ice-cold buffer A (20 mM Tris-HCl [pH 7.5], 150 mM NaCl, 50 mM NaF, 1 mM Na_3_VO_4_, and 1 mM phenylmethylsulfonyl fluoride). After rapid freezing in liquid nitrogen and thawing in a water bath, cells were centrifuged at 16,000 × *g* for 10 min at 4°C. Supernatants were transferred to fresh tubes and were used as the cytosolic fraction. The cell pellet was washed with buffer A and then lysed with buffer A containing 1% (v/v) Triton™ X-100, and centrifuged at 16,000 × *g* for 10 min at 4°C. These supernatants were transferred to fresh tubes and used as the plasma membrane fraction. Cytosolic and plasma membrane fractions were separated by SDS-PAGE and analyzed by Western blotting.

### Flow cytometry

Cells were fixed with 3.7% (w/v) paraformaldehyde for 15 min and then either permeabilized with 0.5% (v/v) Triton™ X-100 in PBS for 10 min or left unpermeabilized. Cells were then incubated with an antibody against MT1-MMP at 2 μg/ml in PBS containing 2% (w/v) BSA for 1 h. After two washes, cells were incubated with Alexa Fluor^®^ 647-labeled donkey anti-rabbit antibody (Life Technologies, Carlsbad, CA, USA) at 1:500 dilution for 2 h. After two washes, cells in PBS were analyzed by flow cytometry. Fluorescence was quantified using a FACSAria™ II Automated Flow Cytometry Sorter (BD Biosciences). Data were analyzed using FACSDiva™ software and histograms were merged using Photoshop 11.0 without any other image manipulation.

### Transwell invasion assays

Transwell inserts with 8 μm pore size were coated with 10 μg of type I collagen at 37°C overnight and the cells were placed in the upper chamber. RPMI 1640 supplemented with 10% (v/v) FBS was added to the lower chamber as a chemoattractant. In some experiments, 5 μM GM6001 (Millipore) was added to both chambers to prevent MMP-dependent cell invasion. Non-migrating or non-invading cells were removed using a cotton swab. Cells were fixed, stained with 0.1% (w/v) crystal violet, and quantified by light microscopy.

### Gelatin zymography assay

Cells were seeded in a 35-mm dish and incubated in RPMI 1640 supplemented with 10% (v/v) FBS for 24 h. Cells were then washed and serum-free RPMI 1640 added. After incubation for 24 or 48 h, the conditioned medium was centrifuged to remove cellular debris and was normalized to cell number prior to electrophoresis on 10% (v/v) polyacrylamide gels with 1 mg/ml gelatin (Sigma-Aldrich). After SDS-PAGE, the gels were washed for 1 h with washing buffer (10 mM Tris-HCl [pH 8.0] and 2.5% [v/v] Triton™ X-100), rinsed with distilled water twice for 10 min, and then incubated for 20 h in developing buffer (50 mM Tris-HCl [pH 7.5], 5 mM CaCl_2_, 0.02% [w/v] NaN_3_). Gels were stained with 0.5% (w/v) Coomassie Blue R-250 for 30 min and destained until the gelatinolytic bands were observed.

### Generation of spheroids

Cell aggregation was induced by growing cell suspensions in 1.5% agarose-coated 48 well plates. After 24 h, 50 μg/ml of collagen I solution was added to promote spheroid formation, and cells were further incubated for 24 h. Subsequently, 1 mg/ml of collagen mixture was poured into the wells, polymerized for 1 h, and then media were added and replaced every 24 h.

### Statistical analysis

Differences between controls and treatments were statistically analyzed by Student's *t*-test (between two groups) or by one-way ANOVA (three or more groups) (PRISM software). Data were expressed as the mean ± standard error of the mean (SEM) of three independent experiments. *p*-values less than 0.05 were considered as statistically significant.

## SUPPLEMENTARY FIGURE



## References

[1] Yilmaz M, Christofori G (2009). EMT, the cytoskeleton, and cancer cell invasion. Cancer Metastasis Rev.

[2] Wang Y, Wen J, Zhang W (2011). MIIP, a cytoskeleton regulator that blocks cell migration and invasion, delays mitosis, and suppresses tumorogenesis. Curr Protein Pept Sci.

[3] Poincloux R, Lizarraga F, Chavrier P (2009). Matrix invasion by tumour cells: a focus on MT1-MMP trafficking to invadopodia. J Cell Sci.

[4] Remacle AG, Rozanov DV, Baciu PC, Chekanov AV, Golubkov VS, Strongin AY (2005). The transmembrane domain is essential for the microtubular trafficking of membrane type-1 matrix metalloproteinase (MT1-MMP). J Cell Sci.

[5] Bartolini F, Gundersen GG (2010). Formins and microtubules. Biochim Biophys Acta.

[6] Wallar BJ, Alberts AS (2003). The formins: active scaffolds that remodel the cytoskeleton. Trends Cell Biol.

[7] Gaillard J, Ramabhadran V, Neumanne E, Gurel P, Blanchoin L, Vantard M, Higgs HN (2011). Differential interactions of the formins INF2, mDia1, and mDia2 with microtubules. Mol Biol Cell.

[8] Li F, Higgs HN (2003). The mouse Formin mDia1 is a potent actin nucleation factor regulated by autoinhibition. Curr Biol.

[9] Chaturvedi LS, Marsh HM, Basson MD (2011). Role of RhoA and its effectors ROCK and mDia1 in the modulation of deformation-induced FAK, ERK, p38, and MLC motogenic signals in human Caco-2 intestinal epithelial cells. Am J Physiol Cell Physiol.

[10] Tsuji T, Ishizaki T, Okamoto M, Higashida C, Kimura K, Furuyashiki T, Arakawa Y, Birge RB, Nakamoto T, Hirai H, Narumiya S (2002). ROCK and mDia1 antagonize in Rho-dependent Rac activation in Swiss 3T3 fibroblasts. J Cell Biol.

[11] Yamana N, Arakawa Y, Nishino T, Kurokawa K, Tanji M, Itoh RE, Monypenny J, Ishizaki T, Bito H, Nozaki K, Hashimoto N, Matsuda M, Narumiya S (2006). The Rho-mDia1 pathway regulates cell polarity and focal adhesion turnover in migrating cells through mobilizing Apc and c-Src. Mol Cell Biol.

[12] Tanji M, Ishizaki T, Ebrahimi S, Tsuboguchi Y, Sukezane T, Akagi T, Frame MC, Hashimoto N, Miyamoto S, Narumiya S (2010). mDia1 targets v-Src to the cell periphery and facilitates cell transformation, tumorigenesis, and invasion. Mol Cell Biol.

[13] Artym VV, Zhang Y, Seillier-Moiseiwitsch F, Yamada KM, Mueller SC (2006). Dynamic interactions of cortactin and membrane type 1 matrix metalloproteinase at invadopodia: defining the stages of invadopodia formation and function. Cancer Res.

[14] Lizarraga F, Poincloux R, Romao M, Montagnac G, Le Dez G, Bonne I, Rigaill G, Raposo G, Chavrier P (2009). Diaphanous-related formins are required for invadopodia formation and invasion of breast tumor cells. Cancer Res.

[15] Bravo-Cordero JJ, Marrero-Diaz R, Megias D, Genis L, Garcia-Grande A, Garcia MA, Arroyo AG, Montoya MC (2007). MT1-MMP proinvasive activity is regulated by a novel Rab8-dependent exocytic pathway. Embo J.

[16] Wiesner C, Faix J, Himmel M, Bentzien F, Linder S (2010). KIF5B and KIF3A/KIF3B kinesins drive MT1-MMP surface exposure, CD44 shedding, and extracellular matrix degradation in primary macrophages. Blood.

[17] Gasman S, Kalaidzidis Y, Zerial M (2003). RhoD regulates endosome dynamics through Diaphanous-related Formin and Src tyrosine kinase. Nat Cell Biol.

[18] Kessenbrock K, Plaks V, Werb Z (2010). Matrix metalloproteinases: regulators of the tumor microenvironment. Cell.

[19] Lin Y, Chang G, Wang J, Jin W, Wang L, Li H, Ma L, Li Q, Pang T (2011). NHE1 mediates MDA-MB-231 cells invasion through the regulation of MT1-MMP. Exp Cell Res.

[20] Itoh Y, Takamura A, Ito N, Maru Y, Sato H, Suenaga N, Aoki T, Seiki M (2001). Homophilic complex formation of MT1-MMP facilitates proMMP-2 activation on the cell surface and promotes tumor cell invasion. Embo J.

[21] Lee H, Chang KW, Yang HY, Lin PW, Chen SU, Huang YL (2013). MT1-MMP regulates MMP-2 expression and angiogenesis-related functions in human umbilical vein endothelial cells. Biochem Biophys Res Commun.

[22] Gopinath SD, Narumiya S, Dhawan J (2007). The RhoA effector mDiaphanous regulates MyoD expression and cell cycle progression via SRF-dependent and SRF-independent pathways. J Cell Sci.

[23] DeWard AD, Eisenmann KM, Matheson SF, Alberts AS (2010). The role of formins in human disease. Biochim Biophys Acta.

[24] Wickstrom SA, Lange A, Hess MW, Polleux J, Spatz JP, Kruger M, Pfaller K, Lambacher A, Bloch W, Mann M, Huber LA, Fassler R (2010). Integrin-linked kinase controls microtubule dynamics required for plasma membrane targeting of caveolae. Dev Cell.

[25] Zaoui K, Honore S, Isnardon D, Braguer D, Badache A (2008). Memo-RhoA-mDia1 signaling controls microtubules, the actin network, and adhesion site formation in migrating cells. J Cell Biol.

[26] Steffen A, Le Dez G, Poincloux R, Recchi C, Nassoy P, Rottner K, Galli T, Chavrier P (2008). MT1-MMP-dependent invasion is regulated by TI-VAMP/VAMP7. Curr Biol.

[27] Bananis E, Murray JW, Stockert RJ, Satir P, Wolkoff AW (2000). Microtubule and motor-dependent endocytic vesicle sorting in vitro. J Cell Biol.

[28] Radichev IA, Remacle AG, Sounni NE, Shiryaev SA, Rozanov DV, Zhu W, Golubkova NV, Postnova TI, Golubkov VS, Strongin AY (2009). Biochemical evidence of the interactions of membrane type-1 matrix metalloproteinase (MT1-MMP) with adenine nucleotide translocator (ANT): potential implications linking proteolysis with energy metabolism in cancer cells. Biochem J.

[29] Dompierre JP, Godin JD, Charrin BC, Cordelieres FP, King SJ, Humbert S, Saudou F (2007). Histone deacetylase 6 inhibition compensates for the transport deficit in Huntington's disease by increasing tubulin acetylation. J Neurosci.

[30] Reed NA, Cai D, Blasius TL, Jih GT, Meyhofer E, Gaertig J, Verhey KJ (2006). Microtubule acetylation promotes kinesin-1 binding and transport. Curr Biol.

[31] Wen Y, Eng CH, Schmoranzer J, Cabrera-Poch N, Morris EJ, Chen M, Wallar BJ, Alberts AS, Gundersen GG (2004). EB1 and APC bind to mDia to stabilize microtubules downstream of Rho and promote cell migration. Nat Cell Biol.

[32] Kenny PA, Lee GY, Myers CA, Neve RM, Semeiks JR, Spellman PT, Lorenz K, Lee EH, Barcellos-Hoff MH, Petersen OW, Gray JW, Bissell MJ (2007). The morphologies of breast cancer cell lines in three-dimensional assays correlate with their profiles of gene expression. Mol Oncol.

[33] Liu J, Tan Y, Zhang H, Zhang Y, Xu P, Chen J, Poh YC, Tang K, Wang N, Huang B (2012). Soft fibrin gels promote selection and growth of tumorigenic cells. Nat Mater.

[34] Chen K, Huang YH, Chen JL (2013). Understanding and targeting cancer stem cells: therapeutic implications and challenges. Acta Pharmacol Sin.

[35] Friedrich J, Seidel C, Ebner R, Kunz-Schughart LA (2009). Spheroid-based drug screen: considerations and practical approach. Nat Protoc.

[36] Charoen KM, Fallica B, Colson YL, Zaman MH, Grinstaff MW (2014). Embedded multicellular spheroids as a biomimetic 3D cancer model for evaluating drug and drug-device combinations. Biomaterials.

[37] Sahlberg SH, Spiegelberg D, Glimelius B, Stenerlow B, Nestor M (2014). Evaluation of cancer stem cell markers CD133, CD44, CD24: association with AKT isoforms and radiation resistance in colon cancer cells. PLoS One.

[38] Zhu Z, Hao X, Yan M, Yao M, Ge C, Gu J, Li J (2010). Cancer stem/progenitor cells are highly enriched in CD133+CD44+ population in hepatocellular carcinoma. Int J Cancer.

[39] Jiang W, Zhang Y, Kane KT, Collins MA, Simeone DM, di Magliano MP, Nguyen KT (2015). CD44 regulates pancreatic cancer invasion through MT1-MMP. Mol Cancer Res.

[40] Remacle A, Murphy G, Roghi C (2003). Membrane type I-matrix metalloproteinase (MT1-MMP) is internalised by two different pathways and is recycled to the cell surface. J Cell Sci.

[41] Williams KC, Coppolino MG (2011). Phosphorylation of membrane type 1-matrix metalloproteinase (MT1-MMP) and its vesicle-associated membrane protein 7 (VAMP7)-dependent trafficking facilitate cell invasion and migration. J Biol Chem.

[42] Kitzing TM, Sahadevan AS, Brandt DT, Knieling H, Hannemann S, Fackler OT, Grosshans J, Grosse R (2007). Positive feedback between Dia1, LARG, and RhoA regulates cell morphology and invasion. Genes Dev.

[43] Clark ES, Whigham AS, Yarbrough WG, Weaver AM (2007). Cortactin is an essential regulator of matrix metalloproteinase secretion and extracellular matrix degradation in invadopodia. Cancer Res.

[44] Li Z, Xu Y, Zhang C, Liu X, Jiang L, Chen F (2013). Mammalian diaphanous-related formin 1 is required for motility and invadopodia formation in human U87 glioblastoma cells. Int J Mol Med.

[45] Caldieri G, Capestrano M, Bicanova K, Beznoussenko G, Baldassarre M, Buccione R (2012). Polarised apical-like intracellular sorting and trafficking regulates invadopodia formation and degradation of the extracellular matrix in cancer cells. Eur J Cell Biol.

[46] Revach OY, Weiner A, Rechav K, Sabanay I, Livne A, Geiger B (2015). Mechanical interplay between invadopodia and the nucleus in cultured cancer cells. Sci Rep.

[47] Even-Ram S, Yamada KM (2005). Cell migration in 3D matrix. Curr Opin Cell Biol.

[48] Wu Y, Guo X, Brandt Y, Hathaway HJ, Hartley RS (2011). Three-dimensional collagen represses cyclin E1 via beta1 integrin in invasive breast cancer cells. Breast Cancer Res Treat.

[49] Tibbitt MW, Anseth KS (2009). Hydrogels as extracellular matrix mimics for 3D cell culture. Biotechnol Bioeng.

[50] Wolf K, Friedl P (2009). Mapping proteolytic cancer cell-extracellular matrix interfaces. Clin Exp Metastasis.

[51] Gardberg M, Kaipio K, Lehtinen L, Mikkonen P, Heuser VD, Talvinen K, Iljin K, Kampf C, Uhlen M, Grenman R, Koivisto M, Carpen O (2013). FHOD1, a formin upregulated in epithelial-mesenchymal transition, participates in cancer cell migration and invasion. PLoS One.

[52] Zeng Y, Xie H, Qiao Y, Wang J, Zhu X, He G, Li Y, Ren X, Wang F, Liang L, Ding Y (2015). Formin-like2 regulates Rho/ROCK pathway to promote actin assembly and cell invasion of colorectal cancer. Cancer Sci.

[53] LaBarge MA (2010). The difficulty of targeting cancer stem cell niches. Clin Cancer Res.

[54] Huang J, Nakamura K, Ito Y, Uzuka T, Morikawa M, Hirai S, Tomihara K, Tanaka T, Masuta Y, Ishii K, Kato K, Hamada H (2005). Bcl-xL gene transfer inhibits Bax translocation and prolongs cardiac cold preservation time in rats. Circulation.

[55] Clancy SM, Chen B, Bertaso F, Mamet J, Jegla T (2009). KCNE1 and KCNE3 beta-subunits regulate membrane surface expression of Kv12. 2 K(+) channels in vitro and form a tripartite complex in vivo. PLoS One.

