# Supplementary material for: mDia1 regulates breast cancer invasion by controlling membrane type 1-matrix metalloproteinase localization
Source: Oncotarget. 2016 Feb 17;7(14):17829–43. doi: 10.18632/oncotarget.7429 (PMC4951253; doi:10.18632/oncotarget.7429)
Supplement: Supplementary file 1 [file oncotarget-07-17829-s001.pdf]

SUPPLEMENTARY FIGURE

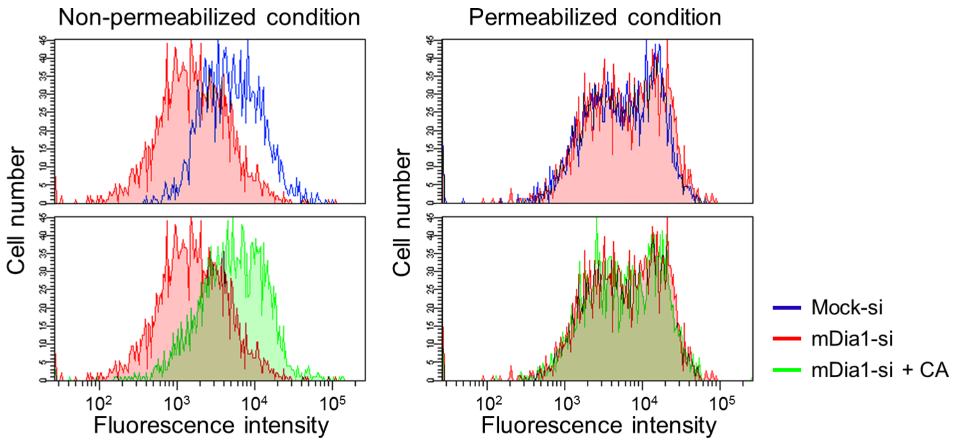

Supplementary Figure S1. Localization of cell surface MT1-MMP is regulated by mDia1. Flow cytometry analysis was performed for permeabilized and non-permeabilized samples to quantify total and membrane-associated MT1-MMP, respectively. Cells transfected with mock siRNA (Mock-si), mDia1 siRNA (mDia1-si) plus GFP, or mDia1 siRNA plus GFP-tagged mDia1-CA plasmid (mDia1-si + CA) were incubated for 24 h, then fixed and stained with an antibody against MT1-MMP, followed by an Alexa Fluor® 647-labeled secondary antibody. GFP-positive cells were first sorted and then, the fluorescence of Alexa Fluor® 647 was quantified by flow cytometry.
